# Periostin Expression and Its Prognostic Value for Colorectal Cancer

**DOI:** 10.3390/ijms160612108

**Published:** 2015-05-27

**Authors:** Zewu Li, Xin Zhang, Yongmei Yang, Sanhui Yang, Zhaogang Dong, Lutao Du, Lili Wang, Chuanxin Wang

**Affiliations:** 1 Department of Clinical Laboratory, Qilu Hospital, Shandong University, Jinan 250012, China; E-Mails: lizwlgs@yeah.net (Z.L.); zhangxin21@126.com (X.Z.); yymxx@126.com (Y.Y.); dongzg790828@gmail.com (Z.D.); dulutao1984@163.com (L.D.); wanglili808@163.com (L.W.); 2 Department of General Surgery, Qilu Hospital, Shandong University, Jinan 250012, China; E-Mail: yangsanhui1234@163.com

**Keywords:** colorectal cancer, POSTN, immunohistochemistry, survival analysis

## Abstract

Integrin is important for cell growth, invasion and metastasis, which are frequently observed in malignant tumors. The periostin (POSTN) gene encodes the ligand for integrin, one of the key focal adhesion proteins contributing to the formation of a structural link between the extracellular matrix and integrins. High expression levels of the POSTN gene are correlated with numerous human malignancies. We examined POSTN protein in colorectal cancer specimens from 115 patients by strictly following up using immunohistochemistry. Cytoplasm immunohistochemical staining showed POSTN protein expression in colorectal cancers. The positive expression rate of POSTN protein (59.13%, 68/115) in colorectal cancers was significantly higher than that in adjacent normal colon mucosa (0.47%, 11/109). POSTN over-expression in colorectal cancers was positively correlated with tumor size, differentiation, lymph node metastasis, serosal invasion, clinical stage and five-year survival rates. Further analysis showed that patients with advanced stage colorectal cancer and high POSTN expression levels had lower survival rates than those with early stage colorectal cancer and low POSTN expression levels. Overall, our results showed that POSTN played an important role in the progression of colorectal cancers.

## 1. Introduction

Colorectal cancer (CRC) is one of the most common malignancies worldwide, with about 1.2 million new cases and 608,700 deaths every year [1]. Colorectal carcinogenesis is a multistep process involving apoptosis, differentiation and survival mechanisms [2]. About half of the individuals with locally advanced CRC can be cured by surgery and multimodal treatment. Because traditional methods do not allow precise prediction of prognosis for the patients after surgical removal of the primary tumor, there is an urgent need for biomarkers capable of distinguishing patients with poor or good prognoses [3]. Although current novel monoclonal antibody-based therapies have improved the prognosis of colorectal cancer patients, a significant proportion of patients still die from the disease, and the clinical outcome and prognosis of colorectal cancer patients remains poor [4,5]. Consequently, to provide better treatment strategies, there is an urgent need to further understand the precise molecular mechanism of CRC and to identify new prognostic biomarkers and therapeutic targets for colorectal cancer.

Periostin (POSTN), a member of the fasciclin domain or osteoblast-specific factor 2 (OSF-2) family of proteins, contains a signal peptide coding sequence (*N*-terminal) and four repeat structures (RDS). Each repeat structure has two fasciclin I domains that are highly conserved to a type of cell-cell adhesion protein expressed in the insect nervous system [6,7]. It is secreted mainly by osteoblasts and their precursor cells in the periosteum and the periodontal membrane, and can promote the proliferation and differentiation of osteoblasts and periosteai bone precursor cells for aggregation and adhesion [6]. POSTN functions as a multidomain adaptor protein that integrates multiple signals such as cell surface receptors, integrins and growth factors [8]. Through these protein-protein interactions, it regulates a variety of physiological processes including cell motility, metastasis, matrix organization, tissue remolding, cell proliferation and survival [9]. Recently, it has been revealed that POSTN is overexpressed in various human cancers [10], including neuroblastoma [11], as well as head and neck [12], nasopharyngeal [13], thyroid [14], oral [15], breast [16] and ovarian cancers [9]. Bao *et al.* have reported that in CRC, periostin potently promotes metastatic growth of colon cancer by augmenting cell survival via the Akt/PKB pathway [17]. More recently, Wu *et al.* have demonstrated that periostin is related to the liver metastasis of CRC and may be a potential target for CRC [18]. However, the clinical-pathological implications of POSTN in CRC are lacking.

To investigate its prognostic value, we selected 115 cases of colorectal cancer and investigated the expression of POSTN by immunohistochemical staining. Our data showed that POSTN was frequently upregulated in colorectal cancers. These findings suggest that POSTN might be a predictor for poor prognosis in patients with colorectal cancer.

## 2. Results

### 2.1. POSTN Protein Is Over-Expressed in Colorectal Cancer

**Figure 1:**
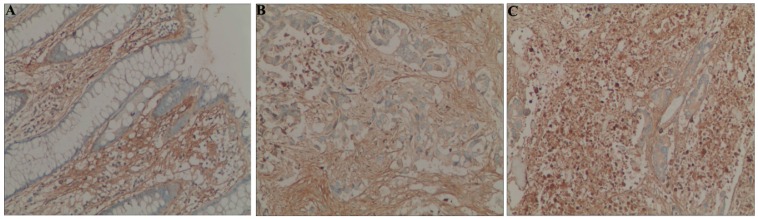
Immunohistochemical staining of POSTN protein in colorectal cancer and normal mucosa cells (original magnification, ×200). (**A**) POSTN is negative in normal colorectal mucosa; (**B**) Low levels of POSTN expression in colorectal cancer cells; and (**C**) High levels of POSTN expression in colorectal cancer cells.

Immunohistochemical staining revealed that the POSTN protein had a cytoplasmic expression pattern in colorectal cancer cells (Figure 1). A low level of POSTN protein expression was detected in 93.13% (108/115) of colorectal cancer cells, which was significantly higher than in adjacent normal colon mucosa (31.30%, 36/115) cells. Similarly, a high level of POSTN protein expression was detected in 59.13% (68/115) of colorectal cancers cells, which was significantly higher than that in adjacent normal colon mucosa (10.47%, 11/109) (*p* < 0.01).

### 2.2. Relationship between Clinical-Pathological Characteristics and POSTN Expression in Colorectal Cancer Tissues

To evaluate the relationship between POSTN protein expression and colorectal cancer progression, we further evaluated the correlation between POSTN expression and clinical-pathological characteristics. The data presented in Table 1 show that a high level of POSTN protein expression was significantly correlated with tumor size (*p* = 0.014), differentiation (*p* = 0.01), lymph nodes metastasis (*p* = 0.006), serosal invasion (*p* = 0.0003) and TNM stages (*p* = 0.0002). However, the over expression of POSTN protein was not related to gender, age or tumor location. Taken together, these results suggest that a high level of POSTN expression might be related to colorectal cancer progression.

**Table 1:** POSTN expression and clinical-pathological characteristics in colorectal cancer patients (*n* = 115).

Characteristic	No. of Cases	High Expression Cases (%)	OR (95% CI)	*p* Value
Gender			1.451 (0.594–3.539)	0.412
Male	90	55 (61.11%)		
Female	25	13 (52%)		
Age (years old)			0.669 (0.329–1.359)	0.265
<53	59	29 (49.15%)		
≥53	56	39 (69.64%)		
Location			0.520 (0.244–1.109)	0.089
Colon	60	31 (51.67%)		
Rectum	55	37 (67.27%)		
Tumor size (cm)			0.384 (0.177–0.830)	0.014
<5	60	29 (48.33%)		
≥5	55	39 (70.90%)		
Differentiation			0.370 (0.172–0.797)	0.010
Well	52	24 (46.15%)		
Poorly	63	44 (69.84%)		
Serosal invasion			0.237 (0.106–0.529)	0.0003
−	60	26 (43.33%)		
+	55	42 (76.36%)		
Lymph node metastasis			0.335 (0.152–0.736)	0.006
−	63	30 (47.62%)		
+	52	38 (73.07%)		
Stage			0.226 (0.010–0.510)	0.0002
I–IIA	62	27 (43.55%)		
IIB–IV	53	41 (77.36%)		

### 2.3. Correlation between POSTN Levels and Patient Survival

**Figure 2:**
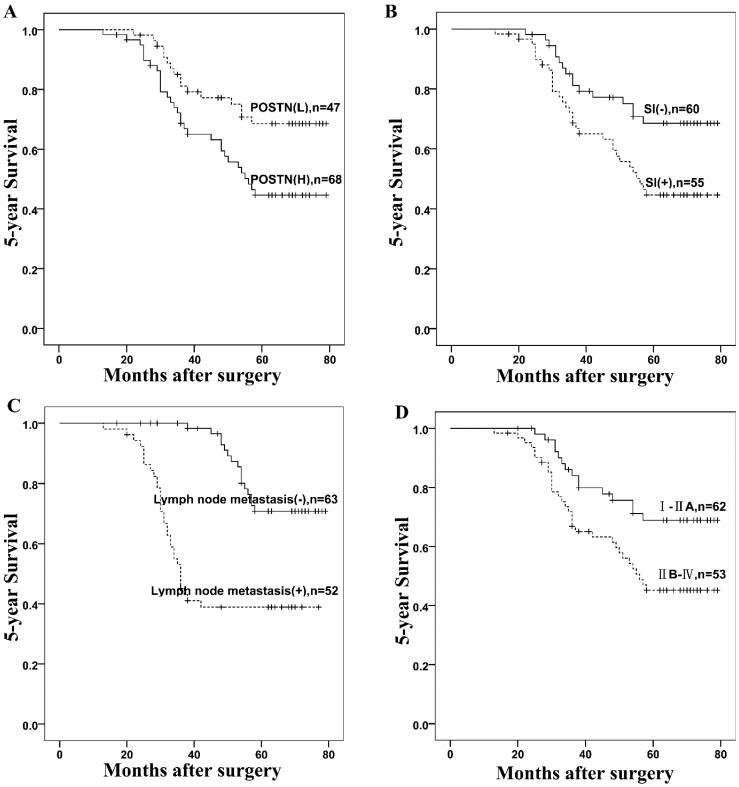
Kaplan-Meier analysis of five-year survival rates in 115 colorectal cancer patients according to (**A**) POSTN expression (*p* = 0.022); (**B**) Serosal invasion (SI) (*p* < 0.001); (**C**) Lymph nodes metastasis (*p* < 0.001); and (**D**) TNM stage (*p* < 0.001). Vertical bars represent censored patients.

To further demonstrate the importance of high POSTN expression in the prognosis of colorectal cancer progression, we analyzed the correlations between POSTN expression and factors associated with the aggressiveness of colorectal cancer. The cumulative 5-year overall survival rate was 59.13%, and the 5-year cumulative probability of survival for patients with high levels of POSTN expression (45.6%) was significantly lower than that for patients with low levels of POSTN expression (78.7%) (*p* = 0.001, Figure 2A). In addition, the results of Kaplan-Meier analysis and log-rank test showed that survival time was also associated with serosal invasion (*p* = 0.014, Figure 2B), lymph nodes metastasis (*p* < 0.001, Figure 2C) and tumor stage (*p* = 0.01, Figure 2D).

### 2.4. Univariate and Multivariate Cox Analysis for Prognosis of Patients with Colorectal Cancer

Using univariate analysis, we found statistically significant correlations between overall survival and POSTN level, serosal invasion, lymph nodes metastasis and tumor stage (Table 2). We found that lymph nodes metastasis (*p* < 0.001) and POSTN expression (*p* = 0.044) were prognostic factors for overall survival rates (Table 3).

**Table 2:** Univariate survival analyses of various factors in patients with colorectal cancer.

Factors	B	SE	Wald	HR	95% CI		*p* Value
					Low	Upper	
Age	0.253	0.292	0.75	0.777	0.438	1.376	0.386
Gender	0.159	0.345	0.212	1.172	0.596	2.303	0.645
Tumor size	0.139	0.292	0.225	1.149	0.648	2.035	0.635
Location	0.149	0.292	0.261	1.161	0.655	2.057	0.609
Differentiation	0.174	0.295	0.349	0.84	0.471	1.498	0.555
Serosal invasion	0.736	0.308	5.708	0.51	0.262	0.876	0.017
Lymph node metastasis	1.363	0.311	19.268	3.909	2.127	7.185	<0.001
Tumor stage	0.784	0.313	6.259	0.457	0.247	1.844	0.012
POSTN	1.17	0.357	10.71	3.222	1.599	6.492	0.001

B: Coefficient; SE: stand error; HR: hazard ratio.

**Table 3:** Multivariant survival analyses of various factors in patients with colorectal cancer.

Factors	B	SE	Wald	HR	95% CI		*p* Value
					Low	Upper	
Lymph node metastasis	1.125	0.321	12.319	3.08	1.643	5.774	<0.001
Serosal invasion	0.279	0.372	0.564	0.756	0.365	1.568	0.453
Tumor stage	0.279	0.479	0.543	0.756	0.401	0.36	1.59
POSTN	0.705	0.381	3.427	2.023	0.959	4.266	0.044

## 3. Discussion

Periostin, also called osteoblast-specific factor 2, is a 93.3-kD secreted protein and can promote integrin dependent cell adhesion and motility [19,20]. Periostin-activated signaling pathways promote cellular survival, angiogenesis and resistance to hypoxia induced cell death. Additionally, periostin is upregulated in response to the stress of hypoxia in the human A549 non-small cell lung cancer cell line and in rat pulmonary arterial smooth muscle cells (PASMCs) [17,21]. It has been frequently reported lately that POSTN is overexpressed in various types of human malignant tumors. For instance, Qiu *et al.* have reported that periostin is a hypoxia-response gene and mediates a cross talk between GC and endothelial cells under hypoxia, partially through the regulation of VEGF expression [22]. Wong *et al*. have shown that POSTN may be a biomarker of the esophageal tumor microenvironment that can be used to detect pre neoplastic lesions [23]. Lee *et al.* have found that mesenchymal stem cell-derived TGFBI and periostin play key roles in tumorigenesis by stimulating adhesion of prostate cancer cells [24]. Increase in COMP and periostin expression and decrease in VAP-1 expression in prostate have been shown to be associated with aggressive prostate cancer [25]. Periostin expression has also been correlated with the increase of Gleason score and the aggressiveness of prostate diseases [26]. However, the downregulation of POSTN mRNA is significantly related to high grade bladder cancer [27,28]. Furthermore, Kanno *et al*. have demonstrated that POSTN has biphasic effects on the migration of pancreatic carcinoma [29]. These results suggest that POSTN may have different functions for different pathological types of cancer, and POSTN expression level and its relationship with prognosis in colorectal cancer have not been elucidated.

In the present study, we performed immunohistochemical staining of POSTN protein and survival data analyses in colorectal cancer cells and their adjacent normal tissue counterparts in 115 cases. We found that the expression levels of the POSTN oncoprotein were significantly higher in colorectal cancers than in adjacent normal tissues. These findings indicated that POSTN potentially played important roles in the progression of colorectal cancer. Results of previous studies have also provided some indirect evidence. For example, circulating periostin may help identify patients with more aggressive colorectal cancer [30]. Because POSTN may be present at higher levels in immature cells than in differentiated counterparts, it can also aid in gauging the differentiation potential of tumor cells. Liu *et al*. [31] have reported that periostin is a nicotine target gene in gastric cancer and play roles in gastric cancer cell growth, invasion, drug resistance, and EMT facilitated by nicotine. Lee *et al.* [32] have shown that the periostin-integrin signaling regulates breast cancer progression at multiple levels in tumor cells and the tumor microenvironment. Also, they reported that DNA aptamers targeting periostin can be used to inhibit breast cancer progression. Xu *et al.* have found that periostin is an independent prognostic factor for breast cancer or a potential target for breast cancer [33]. Combined evaluation of CTHRC1 and periostin can serve as a potential marker for breast cancer bone metastasis [34]. These results indicate that POSTN may be an attractive molecular target for therapy.

Here we demonstrated that high POSTN expression was associated with serosal invasion, lymph node metastasis, tumor size and differentiation, which were crucial histological features associated with poor prognosis in colorectal cancer. However, the high expression level of POSTN was not correlated with gender, age or tumor location. Colorectal cancer exhibiting serosal invasion and lymph nodes metastasis had low five-year survival rates. Of particular interest, high POSTN expression was an independent hazard factor in colorectal cancer. These findings suggested that POSTN facilitated not only serosal invasion and lymph node metastasis, but also aggressive cancer behavior, resulting in poor prognosis. Importantly, we found that colorectal cancer with high POSTN expression was correlated with late-stage tumors. To further verify our findings, the sample size will be increased in our future studies.

## 4. Experimental Section

### 4.1. Patients and Sample Collection

A total of 133 CRC patients who underwent radical resection for CRC in the Department of General Surgery, Qilu Hospital of Shandong University between May 2007 and November 2009 were recruited for this study. Of these subjects, 18 patients were excluded because of incomplete follow-up data and 7 patients were excluded because of statistically insignificant distant metastases. The remaining 115 patients had not received preoperative adjuvant therapy and were deemed eligible for the study. All patient data were obtained from clinical and pathologic records, including age, gender, location, tumor size, differentiation, serosal invasion, lymph node metastasis and clinical stage. The tumor location was categorized as colonic in 60 cases and rectal in 55 cases. Staging was evaluated according to the TNM classification of carcinoma of the colon and rectum. From these 115 tumor tissues, 62 were TNM stage I–IIA, which was considered early stage; and 53 samples were stage IIB–IV, an advanced stage according to the Union for International Cancer Control 7th Edition criteria and the World Health Organization classification [35]. Of the 115 cases, 52 were well-differentiated and 63 were poorly differentiated cancers. The resected tumor tissues and paired adjacent non-cancerous tissues (at least 5 cm away from the tumor margin) were collected. This study was approved by the ethics committee of the Qilu Hospital of Shandong University and written informed consent was obtained from each patient or legal representative.

### 4.2. Follow up

The patients were followed up every 3–6 mo after the operation till December 2014, with a median follow-up period of 61 mo (range 13–79 mo). Overall survival (OS) was defined as the period from surgery to death or the end of the study. All data, including physical examination, laboratory results and computed tomography, were collected from hospital records or by patient interviews.

### 4.3. Immunohistochemistry for POSTN in Paraffin-Embedded Tissues

Immunohistochemical analysis was performed using periostin antibody (Santa Cruz Biotechnology, Santa Cruz, CA, USA). Paraffin-embedded CRC tissues were sliced as 5-μm sections, baked at 65 °C for 2 h, and deparaffinized using standard procedures. After antigen retrieval and washing with Tris buffer, the POSTN primary antibody was incubated with the slides and the expression of POSTN was reviewed after development with a peroxidase-conjugated goat anti-rabbit antibody (Zhongshan Golden Bridge Biotechnology, Beijing, China) following the manufacturer’s guidelines.

### 4.4. Evaluation of Immunohistochemical Staining

To avoid discrepancies, a final score was obtained by reassessment on a double-headed microscope. Briefly, the immunostaining for POSTN was semi-quantitatively scored as “−” (negative, no or less than 5% positive cells), “+” (5%–25% weak positive cells), “++” (26%–50% positive cells) and “+++” (more than 50% positive cells). Immunoreactivity was evaluated according to the staining intensity (0: negative, 1: weak, 2: moderate, 3: strong). The total immunoreactive score was calculated as the sum of staining intensity score and the percentage of positive tumor cells score, and graded as follows: −, 0 to 1; +, 2 to 3; ++, 4 to 5; and +++, 6 to 7. POSTN expression level was denoted as high expression (“++” and “+++”) and low expression (“−” and “+”).

### 4.5. Statistical Analysis

Statistical analyses were performed using the SPSS 18.0 software (SPSS Inc., Chicago, IL, USA). Correlation between POSTN expression and clinical-pathological characteristics were evaluated by Chi-square test and Fisher’s exact tests. The survival rates after tumor removal were calculated by the Kaplan-Meier method, and differences in survival curves were analyzed by the Log-rank tests. Multivariate survival analysis was performed on all the significant characteristics measured by univariate survival analysis (gender, age, tumor size, differentiation, lymph node metastasis, serosal invasion, tumor stage, and POSTN expression) through the Cox proportional hazard regression model. Difference with *p* < 0.05 was considered statistically significant.

## 5. Conclusions

In summary, we identified POSTN as a potential biomarker for the evaluation of tumor progression. POSTN expression was commonly seen in cases with poor prognostic factors of colorectal cancer, leading to lymph node metastasis, advanced stage, serosal invasion and reduced survival time. Similar results with an increased sample size would support POSTN as an independent prognostic marker for colorectal cancer. However, further studies are needed to support this hypothesis.
